# Analysis of the Velocity Distribution in Partially-Filled Circular Pipe Employing the Principle of Maximum Entropy

**DOI:** 10.1371/journal.pone.0151578

**Published:** 2016-03-17

**Authors:** Yulin Jiang, Bin Li, Jie Chen

**Affiliations:** 1 College of Mechatronics Engineering and Automation, Shanghai University, Shanghai, People’s Republic of China; 2 College of Electronic Engineering, Jiujiang University, Jiujiang, People’s Republic of China; Nankai University, CHINA

## Abstract

The flow velocity distribution in partially-filled circular pipe was investigated in this paper. The velocity profile is different from full-filled pipe flow, since the flow is driven by gravity, not by pressure. The research findings show that the position of maximum flow is below the water surface, and varies with the water depth. In the region of near tube wall, the fluid velocity is mainly influenced by the friction of the wall and the pipe bottom slope, and the variation of velocity is similar to full-filled pipe. But near the free water surface, the velocity distribution is mainly affected by the contractive tube wall and the secondary flow, and the variation of the velocity is relatively small. Literature retrieval results show relatively less research has been shown on the practical expression to describe the velocity distribution of partially-filled circular pipe. An expression of two-dimensional (2D) velocity distribution in partially-filled circular pipe flow was derived based on the principle of maximum entropy (POME). Different entropies were compared according to fluid knowledge, and non-extensive entropy was chosen. A new cumulative distribution function (CDF) of partially-filled circular pipe velocity in terms of flow depth was hypothesized. Combined with the CDF hypothesis, the 2D velocity distribution was derived, and the position of maximum velocity distribution was analyzed. The experimental results show that the estimated velocity values based on the principle of maximum Tsallis wavelet entropy are in good agreement with measured values.

## Introduction

In city drainage, conveying stormwater, wastewater discharge and other occasions, many water treatment parameters need to be determined according to the stage-discharge relationship [1]. Because the circular transmission pipeline is often in a state of partially-filled, the relationship is general determined based on Manning Equation [2, 3]. But more precise relationship requires knowledge of the cross-sectional velocity distribution. Knight et al. [4–6] found that the maximum velocity of partially-filled circular pipe occurs axially below the water surface by experimental research, and the velocity distribution near the water surface is mainly influenced by the secondary flows [7]. Using a stereoscopic particle image velocimetry system, Yoon et al.[8] measured the three-dimensional velocity distribution in partially-filled circular pipe, and the measurement results confirmed the above findings. These studies greatly improved the research of partially-filled pipe flow. However, according to literature search, relatively little work has been shown on the theoretical solutions of partially-filled circular pipe turbulent velocity distribution.

In recent years, based on Shannon entropy[9], the theory of entropy develops rapidly, and has been applied in many areas. Some well-studied generalized entropy, such as network entropy, graph entropy [10–13] and Tsallis entropy [14], can measure the complexity and robust of engineering, These research results provide a very good approach for the research of two-dimensional (2D) velocity distribution in partially-filled pipe flow. Assuming the cumulative distribution function (CDF) is the function of flow depth, Chiu [15] derived a 2D velocity distribution formula with Shannon entropy [9], which represented the observed data reasonably well in rectangular open channel, and the formula was employed in a series of studies in following years [16–22]. Yoon et al. [8] proved that the Chiu’s method [15] is also suitable to partially-filled circular pipe flow through experimental study. However, because Chiu’s method [15] used too many empirical parameters that have little physical meaning, its practical use is limited. In this paper, the principle of maximum entropy (POME) [23,24] was used to analyze the flow velocity distribution. Based on POME, Luo and Singh [25] derived a 2D velocity distribution expression of rectangular open channels using the Tsallis entropy. Though the approach was either superior or comparable to Chui’s method, the application was limited because of a large number of parameters used. Marini et al.[26] put forward a new 2D velocity distribution method of rectangular open channels with Shannon entropy, in which a new CFD was hypothesized, and the method shown advantage over Chiu’s distribution. Cui and Singh [27] proposed another new method for deriving 2D velocity distribution with Tsallis entropy, and the approach can reasonably describe the velocity near the boundary. These methods were all used in rectangular open channels, but these research results proved that it is feasible to analyze the flow velocity distribution by using POME.

The objective of this study is to derive a 2D velocity distribution expression in partially-filled circular pipe based on the principle of maximum entropy, and interpreted the distribution parameters in terms of hydraulic characteristics. The 2D velocity distribution was tested using experimental data.

## Methods

Derivation of an entropy-based velocity distribution in partially-filled circular pipe includes (1) comparison and selection of entropy, (2) definition of the non-extensive Tsallis wavelet entropy, (3) probability distribution model based on POME, (4) hypothesizing CDF function in the 2D case, (5) 2D velocity distribution, (6) location of maximum velocity, (7) experimental measurement

### Comparison and Selection of Entropy

For arbitrary uncertain systems, let *X* as a random variable to represent the system state features, and *p*_*i*_(*i* = 1,2,…,*N*) as its probability distribution function (PDF). Then, the Shannon entropy [9] of *X* is defined as

$$S_{BG}=-k\sum_{i=1}^{W}\left[p(i)\text{ln}\;p(i\right)]$$ (1)

In which *k* = Boltzmann constant, *W* = the total number of samples. Since the Shannon entropy is built on the basis of thermodynamic Boltzmann-Gibbs (B-G) entropy [9], and B-G entropy belongs to extensive entropy. Then, for two independent subsystems *A* and *B* of the system, the Shannon entropy has the following characteristics [28]:

$$S_{BG}(A+B)=S_{BG}(A)+S_{BG}(B)$$ (2)

That is, Shannon entropy has additivity. In partially-filled circular pipe laminar flow, the total velocity information can be regard as the sum of the information contained in each part of fluid. However, in partially-filled pipe turbulent flow, existing secondary currents phenomenon, energy aliasing, and random interaction of the fluid motion etc, the total information of each part fluid is not completely equal to the measured signal. So the application of Shannon entropy is limited in turbulent flow.

Tsallis proposed a generalized form of entropy [14], which can be written in discrete form as

$$S_{q}=k\frac{1-\sum_{i=1}^{W}p(i{)}^{q}}{q-1}\;q\in R_{n}$$ (3)

where *q* = non-extensive parameter, used to describe the extensive degree of the system, *q* < 1 and *q* > 1 represent the super-extensive and sub-extensive characteristics of the system respectively[28,29]. Then, for two independent subsystems *A* and *B* of the system, the Tsallis entropy has the following characteristic

$$S_{q}(A+B)=S_{q}(A)+S_{q}(B)+(1-q)S_{q}(A)S_{q}(B)$$ (4)

Different system can choose different non-extensive parameter to calculate the entropy. When *q* → 1, it’s immediately verified that

$$\underset{\text{q}\to 1}{\text{lim}}S_{q}=k\underset{q\to 1}{\text{lim}}\frac{1-\sum_{i=1}^{W}p(i{)}^{q}}{q-1}=-k\sum_{i=1}^{W}p(i)\text{ln}\;p(i))=S_{BG}$$ (5)

The above analysis shows that the Tsallis entropy is the generalized form of Shannon entropy, which can describe the system with extensive and non-extensive properties. Therefore, the non-extensive Tsallis entropy was used to measure the velocity information in this study.

### Non-extensive Tsallis Wavelet Entropy

Let *W*_*f*_ = {*d(n)*, *n* = 1,2,…,*N*} represent the set of discrete wavelet coefficients of flow velocity *v*(*t*), and *d* is the wavelet coefficients component, and *N* is the total sampling points. Defined a sliding data window inside wavelet coefficients set, in which *w*_*b*_ ∈ *N* is the window width, and *s* ∈ *N* is the sliding step, then the data window can be expressed as

$$Y(m;w_{b},s)=\left\{d(n),n=1+ms,2+ms,\ldots ,w_{b}+ms\right\}$$ (6)

Where *m* = 0,1,2,…,(*N* − *w*_*b*_)/ *s*. Assuming that $v_{k}(m)=\sum_{i=1}^{M_{w}}v_{km}(i)$ is the sum of flow velocity within the data window *Y*, in which *M*_*w*_ is the wavelet coefficient scale, and $v_{km}(i)=\sum_{n=1+ms}^{w_{\text{b}}+ms}{\left(d_{i}(n)\right)}^{2}$. Let *p*_*m*_(*i*) = *v*_*km*_(*i*)/*v*_*k*_(*m*), and the condition $\sum_{i=1}^{M_{w}}p_{m}(i)=1$. Then at the center of the data window, the non-extensive Tsallis wavelet entropy can be expressed as

$$S_{ve}(m)=\frac{1-\sum_{i=1}^{M_{w}}{\left(p_{m}(i)\right)}^{q}}{q-1}\;q\in R_{n}$$ (7)

where *q* represents non-extensive parameter, *R*_*n*_ is real number. Eq **7** can be expressed in continuous form as

$$S_{ve}(m)=\frac{1-\int_{v_{\text{min}}}^{v_{\text{max}}}{\left[p_{m}(v)\right]}^{q}dv}{q-1}\;q\in R_{n}$$ (8)

where *v*_min_, *v*_max_ is the minimum and maximum value of velocity within the data window separately, and *p*_*m*_(*v*) is the PDF of velocity at any scale, and ∫*p*_*m*_(*v*)*dv* = 1. Because the flow velocity *v* is zero in pipe wall, the Tsallis wavelet entropy at the whole pipe cross section can be expressed as

$$S_{ve}=\frac{1-\int_{0}^{v_{\text{max}}}{\left[p(v)\right]}^{q}dv}{q-1}\;q\in R_{n}$$ (9)

where *v*_max_ is the maximum value of *v*(*t*) in pipe cross section, usually below the water surface. The format of Eq 9 is similar to Tsallis entropy, but Eq 9 is to calculate the wavelet coefficient entropy of flow velocity *v*(*t*), and the wavelet coefficient reflects the velocity distribution of the flow rate signal within the data window, so the Tsallis wavelet entropy reflects the velocity distribution on the cross section of the pipeline.

### Probability Distribution Model based on the POME

The information of partially-filled circular pipe flow characteristics should be obtained to apply the POME, and the velocity information can be obtained through the knowledge of fluid mechanics and observations. Chiu [15] and Barbé et al. [30] found that the velocity distribution can be derived only with mass conservation. Then the constraints of density function *p*(*v*) are expressed as [15,25–27]

$$\int_{0}^{v_{\text{max}}}p(v)dv=1$$ (10)

$$\text{and}\;\int_{0}^{v_{\text{max}}}vf(v)dv=\bar{v}$$ (11)

where $\bar{v}$ is the cross-sectional mean velocity. Eq 10 is the constraint of total probability, and the Eq 11 is the constraint of mass conservation. In order to derive *p*(*v*), the entropy *S*_*ve*_ must be maximized in accordance with the POME, subject to Eqs 10 and 11. Then the lagrangian function *G* can be expressed as

$$G=\frac{1}{q-1}[1-\int_{0}^{v_{\text{max}}}f{\left(v\right)}^{q}dv]+\lambda_{1}[\int_{0}^{v_{\text{max}}}p(v)dv-1]+\lambda_{2}[\int_{0}^{v_{\text{max}}}vp(v)dv-\bar{v}]$$ (12)

where *λ*_1_ and *λ*_2_ are Lagrange multipliers. Differentiating Eq 12 with respect to *p*(*v*), results in

$$\frac{\partial G}{\partial p(v)}=\frac{\partial }{\partial p(v)}\left\{\frac{1}{q-1}[1-\int_{0}^{v_{\text{max}}}p{\left(v\right)}^{q}dv]+\lambda_{1}[\int_{0}^{v_{\text{max}}}p(v)dv-1]+\lambda_{2}[\int_{0}^{v_{\text{max}}}vp(v)dv-\bar{v}]\right\}$$ (13)

In accordance with the POME, the derivative is zero, this meaning that ∂*G*/∂*p*(*v*) = 0, which results in the expression of *p*(*v*) as

$$p(v)={\left[\frac{1}{q}(1-\lambda_{1}-\lambda_{2}v)+\lambda_{1}+\lambda_{2}v\right]}^{1/(q-1)}$$ (14)

Substitution of Eq 14 in Eqs 10 and 11 gives the following equations about Lagrange multipliers *λ*_1_ and *λ*_2_

$${\left[\frac{1}{q}(1-\lambda_{1}-\lambda_{2}v_{\text{max}})+\lambda_{1}+\lambda_{2}v_{\text{max}}\right]}^{q/(q-1)}=\lambda_{2}+{\left(\frac{1}{q}-\frac{\lambda_{1}}{q}+\lambda_{1}\right)}^{q/(q-1)}$$ (15)

$$\int_{0}^{v_{\text{max}}}v{\left[\frac{1}{q}(1-\lambda_{1}-\lambda_{2}v)+\lambda_{1}+\lambda_{2}v\right]}^{1/(q-1)}dv=\bar{v}$$ (16)

If the values of non-extensive parameter *q*, *v*_max_ and $\bar{v}$ were given, the value of Lagrange multiplier can obtained by solving Eqs 15 and 16. But the solving process is complex, and there is no direct analytical solution.

### The Cumulative Distribution Function

The CDF was used to establish the relationship between the space domain and the entropy-based probability distribution function in this paper, so CDF must be able to reflect the geometry of the partially-filled circular pipeline and some important characteristics of velocity distribution. Then the CDF must have the following characteristics: (1) continuous and differentiable, (2) defined between 0 and 1, (3) its value on the pipe wall must be 0, and it reaches 1 at the position of *v*_max_, (4) in the vertical axis of the center line, the CDF is a monotonically increasing function from 0 to *v*_max_, and (5) the same as to any vertical axis of the pipe cross-section.

**Fig 1:**
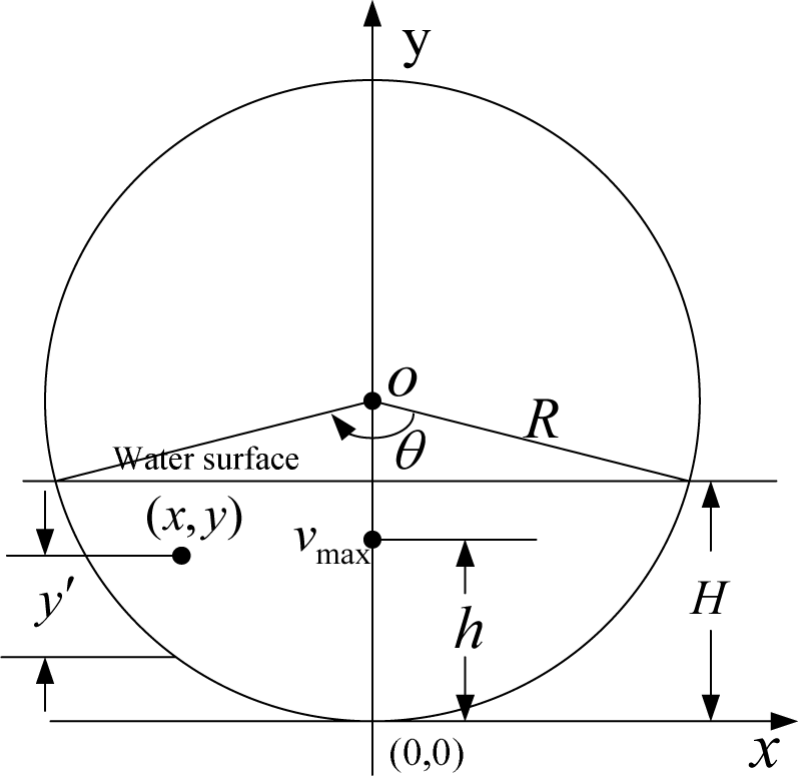
Schematic of Rectangular Coordinate System

An idealized partially-filled circular pipeline channel is shown in Fig 1, in which *R* is the radius, and *H* is the water depth, and *h*(0 ≤ *h* < *H*) is the distance from the position of *v*_max_ to the bottom of pipe. A rectangular coordinate system is set in this way such that the coordinate origin represents the bottom of the circular tube, and *x* presents the transverse distance from the centerline, and *y* presents the vertical depth from *x* axis upward positive, and (*x*, *y*) is a random point inside the pipe.

A CDF should be based on available experimental data and similar relationship in the velocity distribution, so that suitable parameters appear in the final equation depending on *x*, *y* and *R*. The derivation of the CDF involves the following three steps.

The front three characteristics should be first considered. Because the velocity is assumed to zero at the boundary, the corresponding CDF also should be zero, and the simplest equation satisfying the properties(1), (2) and (3) as the parabolic equation

$$F_{1}(v)=1-{\left(\frac{x}{R}\right)}^{2}-{\left(\frac{y}{R}-1\right)}^{2}$$ (17)

At the position of maximum flow velocity, *y* = *h*, which is assumed to occur at the axis passing through the center of water surface, the corresponding CDF *F*_1_(*v*) = 1, then the CDF can be expressed as

$$F_{1}(v)={\left[1-{\left(\frac{y}{h}-1\right)}^{2L}\right]}^{K}\left[1-{\left(\frac{x}{R}\right)}^{D/H}\right]$$ (18)

where *D* is the diameter of the pipeline, when *y* ≤ *h*, *L* = 1 and *K* = 1, while when *y* > *h*, *L* = 2*h* / *H* and *K* = 2(*H* − *h*) / *H*.

Second, the fourth characteristic is considered. At the centerline of pipe cross-section, the CDF increased with the increasing of the flow velocity. So the CDF can be assumed as

$$F_{2}(v)=2y/R-{\left(y/R\right)}^{2}$$ (19)

Because the maximum flow velocity is assumed to occur on or below the water surface, that is to say, when CDF *F*_2_*(v)* = 1, the position of maximum velocity *y*_*v*max_ ≤ *H*, and the *y*_*v*max_ is not the same at different water depths. Then Eq 19 changes to

$$F_{2}(v)=4\left[{\left(y/2R\right)}^{b}-{\left(y/2R\right)}^{2b}\right]$$ (20)

where *b* is a adjustment factor related to water depth. At the position of maximum flow velocity, *y* = *h* and *F*_2_*(v)* = 1, so *b* can be expressed as

b=ln2/[ln(2R)−lnh] (21)

Inserting the expression of *b* into Eq 20 gives

$$F_{2}(v)=4\left[{\left(\frac{y}{2R}\right)}^{\frac{\text{ln}\;2}{\text{ln}(2R)-\text{ln}\;h}}-{\left(\frac{y}{2R}\right)}^{\frac{2\text{ln}\;2}{\text{ln}(2R)-\text{ln}\;h}}\right]$$ (22)

Third, the fifth characteristic is considered. Because the pipe cross section is circular, at any vertical direction, the relative height should be adjusted to

$$y^{\prime }=y-\left(R-\sqrt{R^{2}-x^{2}}\right)$$ (23)

Then the CDF *F*_1_(*v*) and *F*_2_(*v*) also have been changed as

$$F_{1}(v)={\left[1-{\left(\frac{y^{\prime }}{h^{\prime }}-1\right)}^{2L}\right]}^{K}\left[1-{\left(\frac{x}{R}\right)}^{D/H}\right]$$ (24)

$$F_{2}(v)=4\left[{\left(\frac{y^{\prime }}{2R}\right)}^{\frac{\text{ln}\;2}{\text{ln}(2R)-\text{ln}\;h^{\prime }}}-{\left(\frac{y^{\prime }}{2R}\right)}^{\frac{2\text{ln}\;2}{\text{ln}(2R)-\text{ln}\;h^{\prime }}}\right]$$ (25)

where *h*′ represents the vertical distance from maximum velocity to pipe bed in any vertical axis. Integrating Eq 24 and Eq 25, the CDF of the velocity distribution of the partially-filled circular pipe flow can be expressed as

$$F(v)=F_{1}(v)F_{2}(v)=4{\left[1-{\left(\frac{y^{\prime }}{h^{\prime }}-1\right)}^{2L}\right]}^{K}\left[1-{\left(\frac{x}{R}\right)}^{D/H}\right]\left[{\left(\frac{y^{\prime }}{2R}\right)}^{s}-{\left(\frac{y^{\prime }}{2R}\right)}^{2s}\right]$$ (26)

where *s* = ln 2/[ln*(*2*R)* − ln *h*′]

### 2D Velocity Distribution

The next step is to compute velocity distribution with the CDF expressed by Eq 26. Because *v* is the function of *x* and *y*, then *v* can be written as *v*(*x*, *y*), its PDF as *p*[*v*(*x*, *y*)], and the CDF as *F*[*v*(*x*, *y*)]. Referring to the method proposed by Cui and Singh [27], to deal with Eq 14 and Eq 26, the expression of velocity distribution can be obtained as

$$v=\frac{1}{\lambda_{2}}\left\{{\left[\lambda_{1}^{q/(q-1)}+\frac{y^{\prime }\lambda_{2}F(v)}{y}{\left(\frac{q}{q-1}\right)}^{q/(q-1)}\right]}^{1-1/q}-\lambda_{1}\right\}$$ (27)

Eq 27 is the 2D velocity distribution equation in partially-filled circular pipe based on the Tsallis wavelet entropy, and the details of derivation process are shown in Appendix. There exist two Lagrange multipliers *λ*_1_ and *λ*_2_ in Eq 27, which are determined by Eqs 15 and 16, respectively. But, by the above discussion, there is no direct analytical solution for *λ*_1_ and *λ*_2_. To avoid solving these two parameters, following Chiu [15], Cui and Singh [27], we define a dimensionless entropy parameter *M* by

$$M=(q-1)\lambda_{2}v_{\text{max}}/[1+(q-1)(\lambda_{1}+\lambda_{2}v_{\text{max}})]$$ (28)

At the point of maximum velocity, the CDF *F*(*v*) = 1, then the maximum velocity is obtained as

$$v_{\text{max}}=\frac{1}{\lambda_{2}}\left\{{\left[\lambda_{1}^{q/(q-1)}+\frac{y^{\prime }\lambda_{2}}{y}{\left(\frac{q}{q-1}\right)}^{q/(q-1)}\right]}^{1-1/q}-\lambda_{1}\right\}$$ (29)

Substitution of Eqs 28 and 29 in Eq 27 gives the general expression of velocity distribution

$$v=\left(1-\frac{1}{M}\right)v_{\text{max}}+\frac{v_{\text{max}}}{M}{\left\{\frac{y^{\prime }\left[1-{\left(1-M\right)}^{q/(q-1)}\right]F(v)}{y}+{\left(1-M\right)}^{q/(q-1)}\right\}}^{1-1/q}$$ (30)

Eq 30 is the 2D velocity distribution in terms of *M*, *q*, *v*_max_ and 2D CDF. Then, for the cross section of the partially-filled circular pipe, the average velocity can be expressed as

$$\bar{v}=\frac{Q}{S_{A}}=\frac{\int_{S_{A}}vdS_{A}}{S_{A}}=\frac{1}{S_{A}}\int_{S_{A}}\left(1-\frac{1}{M}\right)v_{\text{max}}+\frac{v_{\text{max}}}{M}{\left\{\frac{y^{\prime }\left[1-{\left(1-M\right)}^{q/(q-1)}\right]F(v)}{y}+{\left(1-M\right)}^{q/(q-1)}\right\}}^{1-\frac{1}{q}}dS_{A}$$ (31)

where *Q* is flow discharge; and *S*_*A*_ is flow cross section area.

### Location of Maximum Velocity

In partially-filled circular pipe, the position of maximum velocity is beneath the free surface [8]. But its exact position was difficult to determine due to the secondary currents [31] in the central region [32]. The flow near the wall is significantly affected by the boundary shear and the shape of lateral portion wall. So the position of maximum velocity point is different in each vertical direction. According to the Newton inner friction law, the bed-shear stress is

τ=μ∂v/∂y (32)

where *μ* is the coefficient of kinetic viscosity. And according to the Darcy-Weisbach formula, we obtain

$$\tau =\rho \lambda {\bar{v}}^{2}/8$$ (33)

where *λ* is Darcy friction factor, *ρ* is fluid density. Thus

$$\partial v/\partial y=\rho \lambda {\bar{v}}^{2}/8\mu$$ (34)

On the other hand, by Eq 30, we get

$$\begin{array}{l} \frac{\partial v}{\partial y}=v_{\text{max}}\frac{\left(q-1)[1-{\left(1-M\right)}^{q/\left(q-1\right)}\right]}{Mq}\times \{\frac{y^{\prime }\left[1-{\left(1-M\right)}^{q/(q-1)}\right]F(v)}{y}\\ +{\left(1-M\right)}^{\frac{q}{q-1}}{\}}^{\frac{1}{q}}\times \left[\frac{y^{\prime }F(v)}{y^{2}}+\frac{y^{\prime }}{y}\frac{\partial F(v)}{\partial y}\right] \end{array}$$ (35)

Taking the partial derivatives of Eq 26 with respect to *y*, the expression of ∂*F*(*v*)/∂*y* is obtained

$$\begin{array}{l} \frac{\partial F(v)}{\partial y}=-\frac{8(y^{\prime }-h')}{{\left(h^{\prime }\right)}^{2}}\left[1-{\left(\frac{x}{R}\right)}^{D/H}\right]\left[{\left(\frac{y^{\prime }}{2R}\right)}^{s}-{\left(\frac{y^{\prime }}{2R}\right)}^{2s}\right]\\ +4\left[1-{\left(\frac{x}{R}\right)}^{D/H}\right]{\left[1-{\left(\frac{y^{\prime }}{h^{\prime }}-1\right)}^{2L}\right]}^{K}\left[\frac{s}{2R}{\left(\frac{y^{\prime }}{2R}\right)}^{s-1}-\frac{s}{R}{\left(\frac{y^{\prime }}{2R}\right)}^{2s-1}\right] \end{array}$$ (36)

Substituting Eq 34 in Eq 35, one obtains

$$\begin{array}{l} 1=\frac{8\mu v_{\text{max}}}{\rho \lambda Mq{\bar{v}}^{2}}(q-1)[1-{\left(1-M\right)}^{q/\left(q-1\right)}]\times \{\frac{y^{\prime }\left[1-{\left(1-M\right)}^{q/(q-1)}\right]F(v)}{y}\\ +{\left(1-M\right)}^{\frac{q}{q-1}}{\}}^{\frac{1}{q}}\times \left[\frac{y^{\prime }F(v)}{y^{2}}+\frac{y^{\prime }}{y}\frac{\partial F(v)}{\partial y}\right] \end{array}$$ (37)

Eq 37 can be used to calculate the position of the maximum velocity in any vertical direction. For *x* = 0, *y* = *h*, Eq 37 is simplified as

$$4\left[\frac{s}{2R}{\left(\frac{h}{2R}\right)}^{s-1}-\frac{s}{R}{\left(\frac{h}{2R}\right)}^{2s-1}\right]=\frac{M\rho q\lambda {\bar{v}}^{2}}{8\mu (q-1)v_{\text{max}}\left[1-{\left(1-M\right)}^{q/\left(q-1\right)}\right]}$$ (38)

Eq 38 is the expression of the position of maximum flow velocity on the whole section. Where *λ* is the Darcy friction factor, which can be obtained by Colebrook Equation [33]. Eq 38 yields the depth of maximum velocity of the whole cross section.

### Experimental Measurement

**Fig 2:**
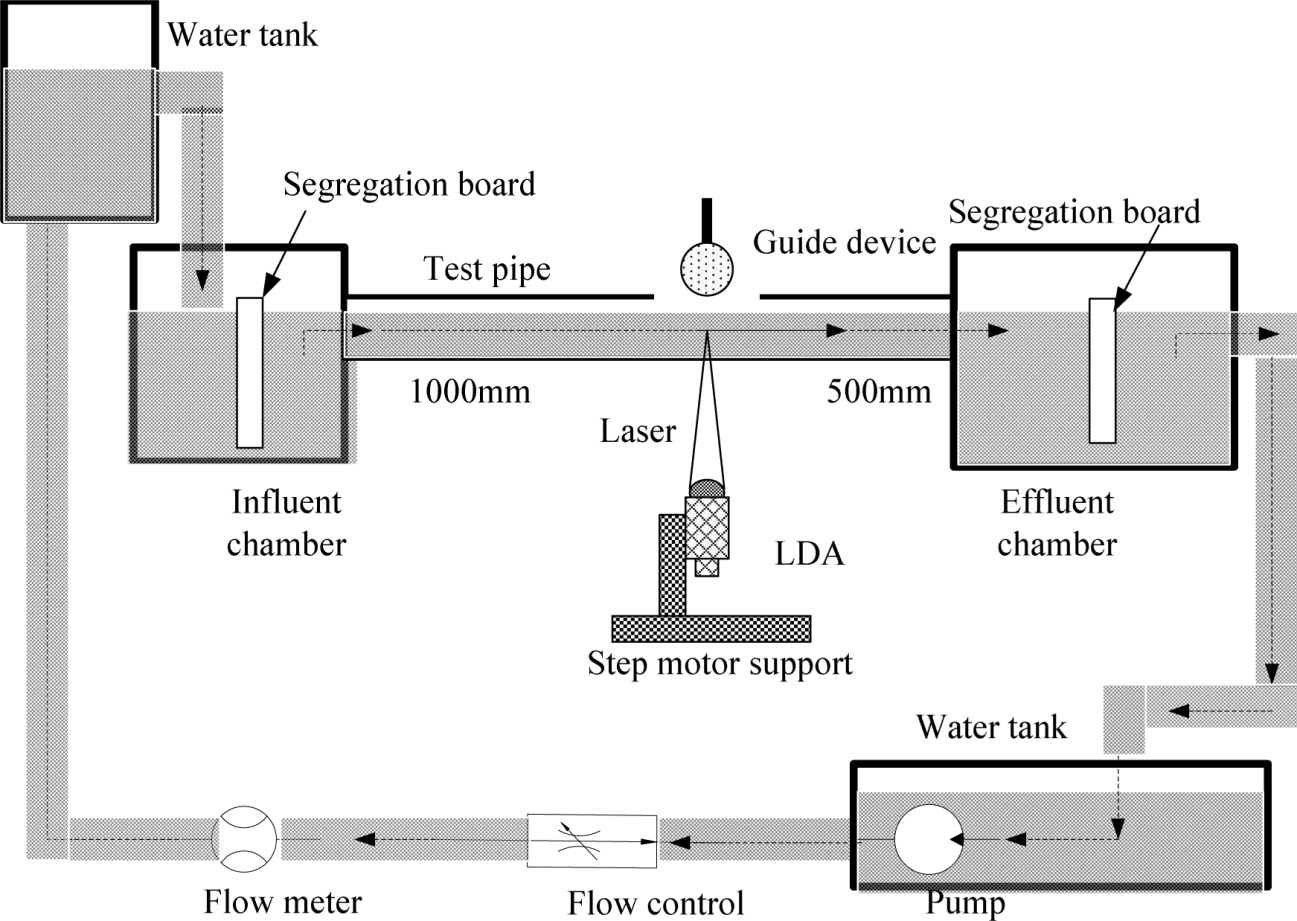
Schematic diagram of experimental apparatus

The test equipment is shown in Fig 2. An accurate electromagnetic flow meter was adopted to measure the mean velocity of fluid, and a Laser Doppler Velocimetry (LDA) was adopted to measure the fluid velocity of single point. The diameter of the transparent acrylic test pipe is *l* = 0.024*m*, and the pipe wall thickness is *L* = 0.003*m*. The distance between the pipe inlet and the observation point is 20*l*, and the distance between the pipe outlet and the measuring point is 15*l*. The hydraulic slope of the test pipe is *S* = 0.0033, the pipe wall was hypothesized to be hydro-dynamically smooth, and the roughness coefficient is *n*_*rc*_ = 0.0085. The refractive index of light in acrylic pipe is *n*_*λ*_ = 1.56 given by instruction book. A laser light emitted form a 30mJ Nd:YAG laser to illuminate the test point, and the laser pulse period is 20*ns*. Some glass spheres of 40 *μm* diameter were injected in order to seed the flow. The glass particles were captured by the LDA.

**Fig 3:**
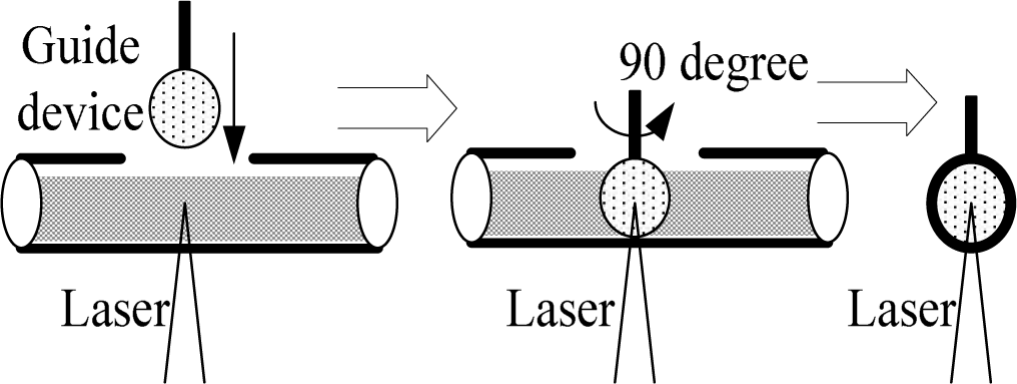
Schematic diagram of guide device

Since the light refractive index is different in acrylic pipe and water, there will be deviation between the measurement position of theoretical and actual. A circular measurement guide device was made, as shown in Fig 3, which the diameter is equal to the test pipe diameter. The measurement point marked on the device, and then LDA measured the velocity in accordance with the mark.

The velocity distribution of different flow depths was measured. The flow depth *H* varies from 30% to 70% of *D*. The hydraulic radius *R*_*h*_ was determined from the flow area *S*_*A*_ and the wetted perimeter *χ* as

$$S_{A}=D^{2}(\theta -\text{sin}\;\theta )/8$$ (39)

χ=Dθ/2 (40)

$$R_{h}=S_{A}/\chi =D(1-\text{sin}\;\theta /\theta )/4$$ (41)

where *θ* is angle between pipe center and free surface.

## Results and Discussion

### Experimental Results

The 2D velocity distribution measured by the LDA system on the cross-sectional plane for 36.2%, 50% and 70% flow depth. Because the flow is turbulent, the position of *v*_max_ below the free surface, and its exact position is difficult to determine. So the central axis velocity distribution function was evaluated by applying a non-linear least-square fit. Then the location of maximum velocity *h* and velocity *v*_max_ can be obtained through the function according to the measured data, in which the velocity of each point was measured ten thousands times, and the average value is taken as the final result.

The values resulted from the least-squares fits are listed in Table 1, for the sake of brevity, only 36.2%, 50% and 70% flow depths are listed. Where the Reynolds, Froude and Weber numbers are defined [8] as $\text{Re}=4\bar{v}R_{h}/\upsilon$, $Fr=\bar{v}/{\left(gD_{m}\right)}^{1/2}$ and $We=\rho {\bar{v}}^{2}R_{h}/\sigma$, respectively, and *υ* is the kinematic viscosity, *g* is the gravitational acceleration, *D*_*m*_ is the hydraulic depth defined as the wet area divided by the free surface width. *σ* is the surface tension. The present flows are turbulent.

**Table 1:** Least-squares fits of velocity distribution function to measured data.

*H/D (%)*	36.2%	50%	70%
*H(mm)*	8.7	12	16.8
*R*_*h*_*(mm)*	4.8	6	7.1
*Re*	3465.9	5029.0	6673.7
*Fr*	0.2568	0.3039	0.3811
*We*	2.20	3.70	5.51
$\bar{v}$ *(m/s)*	0.1816	0.2108	0.2364
*v*_*max*_*(m/s)*	0.2727	0.297	0.3323
*h(mm)*	6.82	8.98	8.32

Obviously, both the mean and maximum velocities gradually increase with the flow depth for the given slope. But the height of maximum velocity is not fixed, the height at 50% depth is higher than 36.2% depth, and the height at 70% depth is lower than 50% depth.

### Comparisons and Discussion

The mean velocity comparison of measured data and Manning formula are listed in Table 2, and the locations of maximum velocity were also compared in this table.

**Table 2:** Comparison of mean velocity and location of maximum velocity.

*H/D (%)*	36.2%	50%	70%
*H(mm)*	8.7	12	16.8
$\bar{v}$_*measure*_*(m/s)*	0.1820	0.2111	0.2362
$\bar{v}$_*manning*_*(m/s)*	0.1816	0.2108	0.2364
*h*_*measure*_ *(mm)*	6.82	8.98	8.32
*h*_*estimate*_*(mm)*	6.85	9.02	8.36

The mean velocity measured by electromagnetic flow meter is very similar to the average velocity obtained from the Manning equation. The locations of maximum velocity estimated with Eq 38 are greater than the measured values, because the estimated values are obtained with hypothetical boundary conditions, and the measured values are obtained under the actual boundary conditions, but error between the two sets of data is very small.

**Fig 4:**
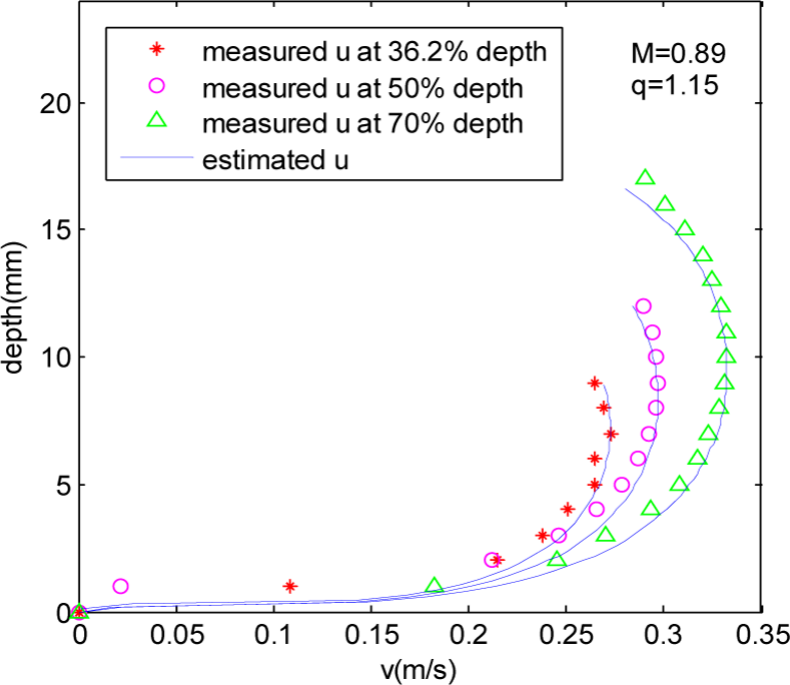
Comparison of measured and estimated axial velocity profiles for 36.2%, 50% and 70% D.

The measured and estimated one-dimensional (1D) axial velocity profiles were compared in Fig 4 for flow depths of 36.2%, 50% and 70%.

According to Fig 4, the estimated data are better consistent with the measured data at different depth, and the maximum velocity occurring below the water surface gradually increase with the flow depth. But in the case of 70%D water depth, there is a big difference between the estimated value and the measured value near the water surface because the gradually contractively tube wall.

**Fig 5:**
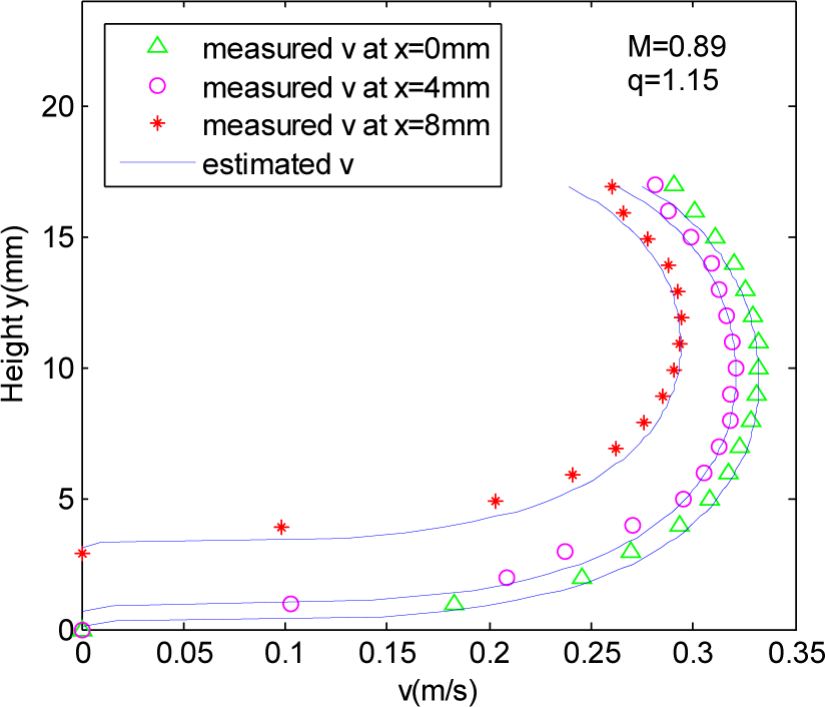
Comparison of one-dimensional velocity distribution at 70% D.

Three different vertical data series of 70% flow depth were also compared, as shown in Fig 5.

In Fig 5, the vertical axis is the relative height from the bed. It is shown in Fig 5 that the velocity increase to maximum and decrease to some value up to the free surface, and the maximum velocity decrease with the distance from the center due to the frictional force by the side wall. At different *x* coordinates, the estimated velocity profile is in good agreement with the measured velocity profile except near the water surface.

**Fig 6:**
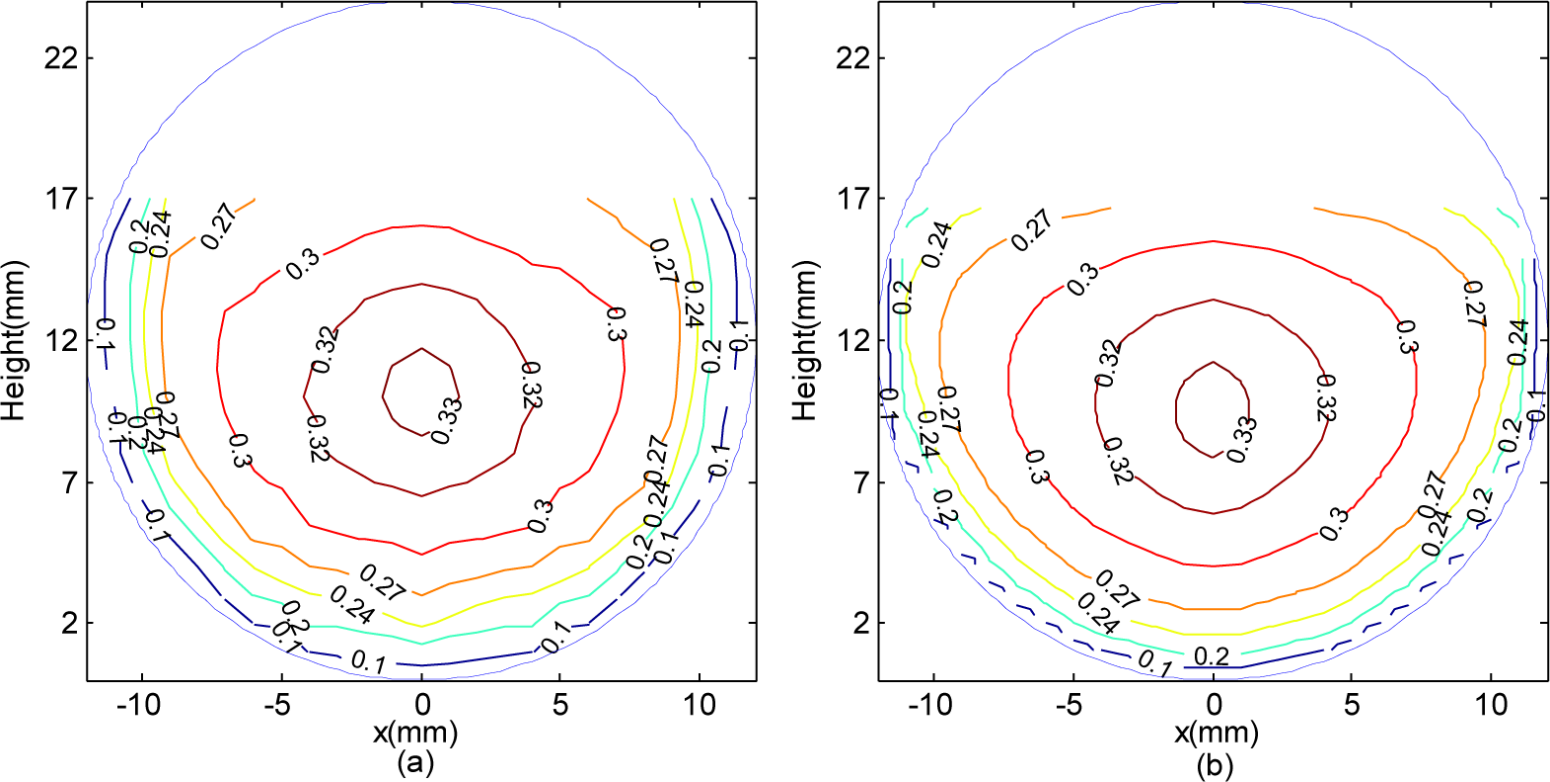
**The contours of 2D velocity distribution**: (a) measured data and (b) estimated data.

The contours of the 2D velocity distribution for the whole cross section are shown in Fig 6(A) and Fig 6(B).

From Fig 6(A), in the proximity of the pipe wall, the flow velocity is smaller due to the friction force of the wall, and the flow velocity is increased with water depth. The farther distance from the tube wall is, the smaller gradient of the velocity is. At the region of pipe center, especially from the water surface to the maximum velocity position, the velocity gradient is less than the gradient from maximum velocity position to pipe wall.

Compare Fig 6(A) with 6(B), below the location of maximum velocity, the velocity distribution of measured value is similar to estimated value. But above the location of maximum velocity, especially near the water surface, the velocity distribution of estimated value is not good match to measured value, due to the flow velocity is mainly influenced by the secondary currents and gradually contractively tube wall. However, the overall trend of estimated velocity distribution contours same as the measured profile. Compared the two contours charts, the estimated values fit the measured values well on the whole.

### Comparison with other Entropy-based Velocity Distributions

**Fig 7:**
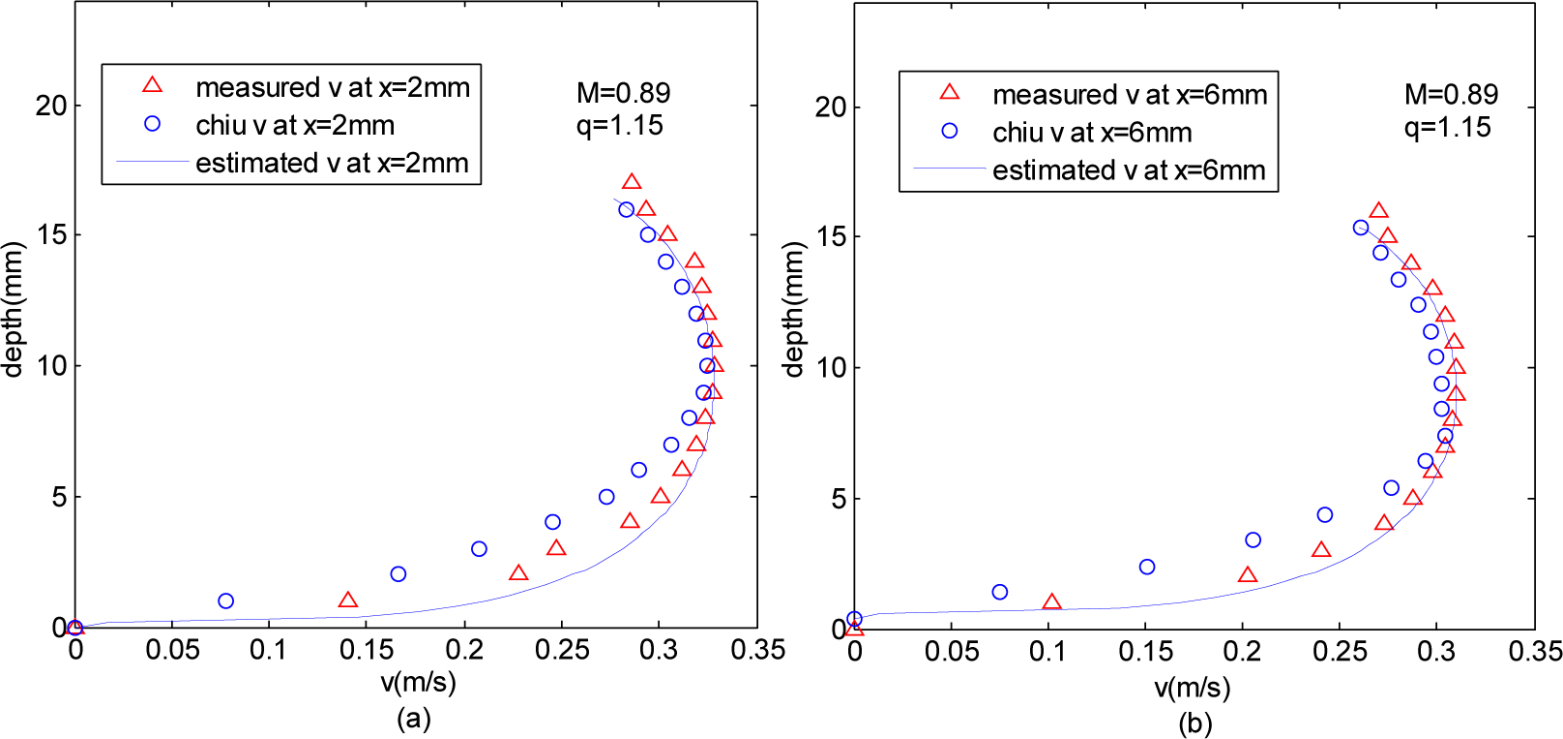
**Comparison of different methods** (a) comparison at *x* = 2*mm* (b) comparison at *x* = 6*mm*.

With the same coordinate system and the same CDF, the profiles based on the proposed velocity distribution were compared with the profiles of Chiu’s method, as Fig 7 shows.

In the central region, both Chiu’s velocity distribution and Tsallis wavelet entropy-based distribution can reflect the measured velocity distribution. But more closer to the lateral wall, for example *x* = 6*mm*, Chiu’s distribution did not capture most of the measured points. It is shown in Table 3 that the two distributions were in good agreement with measured values, with variance no higher than 0.0296*m*^*2*^*/s*^*2*^. The velocity distribution variance based on Tsallis wavelet entropy is larger in the middle region, and smaller near the pipe wall, but Chui’s distribution is just the opposite.

**Table 3:** Variance, in m^2^/s^2^, of Velocity Distribution for Different Methods.

*x*(mm)	Tsallis wavelet entropy-based velocity	Chiu’s(1988) velocity
0	0.0248	0.0170
2	0.0223	0.0155
4	0.0124	0.0205
6	0.0173	0.0262
8	0.0170	0.0296

## Conclusions

This research has derived a new 2D velocity distribution based on the principle of maximum entropy for partially-filled circular pipe flow. The hypothesized CDF can reasonable describe the probability distribution of velocity. On the basis of this assumption, the estimated velocity value based on Tsallis wavelet entropy is very close to the experimental measured value. The estimation of parameters *M* and *q* are determined by mean velocity, maximum velocity and maximum velocity position at two different water depth. Compared with other entropy-based velocity distributions, the velocity distribution method proposed in this paper can reasonably describe the velocity distribution in most region expect near the water surface. Under same boundary conditions, the CDF of velocity distribution have a certain similarity for different hydraulic diameters, so the velocity distribution should also have a certain similarity. The assumption was already verified at 24mm and 50mm diameters pipe by experiments.

In the future, we will continue to optimize the velocity distribution model of partially-filled, and reduce the precondition of solving the model. Also, we intend to employ the model to predict the velocity distribution in partially-filled electromagnetic flow meter. To do so, the influence of hydraulic slope, pipe wall friction and other facts on the velocity distribution must be considered in the model. Hence, this paper can be also seen as a preliminary study for working on the latter problem.

## Appendix

In this appendix, the derivation of the velocity distribution function is presented. We start the derivation from the relation of velocity distribution function and CDF. Taking the partial derivatives of *F*(*v*) with respect to *x* and *y*, one obtains

$$\frac{\partial F(v)}{\partial x}=p(v)\frac{\partial v}{\partial x}=\frac{\partial v}{\partial x}{\left[\frac{1}{q}(1-\lambda_{1}-\lambda_{2}v)+\lambda_{1}+\lambda_{2}v\right]}^{1/(q-1)}$$ (A1)

$$\frac{\partial F(v)}{\partial y}=p(v)\frac{\partial v}{\partial y}=\frac{\partial v}{\partial y}{\left[\frac{1}{t}(1-\lambda_{1}-\lambda_{2}v)+\lambda_{1}+\lambda_{2}v\right]}^{1/(q-1)}$$ (A2)

Now defining a new variable

$$w={\left[\frac{1}{q}+\frac{q-1}{q}\left(\lambda_{1}+\lambda_{2}v\right)\right]}^{\frac{q}{q-1}}$$ (A3)

Taking the partial derivatives of *w* with respect to *x* and *y*, the following equations are obtains

$$\frac{\partial w}{\partial x}=\lambda_{2}{\left[\frac{1}{q}+\frac{q-1}{q}\left(\lambda_{1}+\lambda_{2}v\right)\right]}^{\frac{1}{q-1}}.\frac{\partial v}{\partial x}$$ (A4)

$$\frac{\partial w}{\partial y}=\lambda_{2}{\left[\frac{1}{q}+\frac{q-1}{q}\left(\lambda_{1}+\lambda_{2}v\right)\right]}^{\frac{1}{q-1}}.\frac{\partial v}{\partial y}$$ (A5)

Substitution of Eqs A4 and A5 into Eqs A1 and A2 gives the following relationship between *F*(*v*) and *w*

$$\partial w/\partial x=\lambda_{2}\partial F(v)/\partial x$$ (A6)

$$\partial w/\partial y=\lambda_{2}\partial F(v)/\partial y$$ (A7)

Eqs A6 and A7 can be integrated using the Leibniz rule

$$\int_{\left(0,0\right)}^{\left(x,y\right)}\frac{\partial w}{\partial x}dx+\frac{\partial w}{\partial y}dy=w(x,y)-w(0,0)$$ (A8)

Because the point (0,0) lie on the pipe wall, and *v*(*x*, *y*) at the point is zero, then Eq A8 becomes

$$w(x,y)\;-\;w(0,0)=w(x,y)-{\left[\frac{1}{q}+\frac{q-1}{q}\lambda_{1}\right]}^{\frac{q}{q-1}}$$ (A9)

The definite integral of the left part of Eq A8 is calculated at a generic point (*x*, *y*), which is identified by means of a polygonal curve that start from origin (0,0), pass through the point on the wall $\left(x,R-\sqrt{R^{2}-x^{2}}\right)$, and ends at (*x*, *y*). The CDF is zero at pipe wall below water surface, and the CDF is constantly zero at point (0,0) to $\left(x,R-\sqrt{R^{2}-x^{2}}\right)$, then

$$\begin{array}{l} \int_{\left(0,0\right)}^{\left(x,y\right)}\frac{\partial w}{\partial x}dx+\frac{\partial w}{\partial y}dy=\int_{\left(0,0\right)}^{\left(x,y\right)}\lambda_{2}\frac{\partial F(v)}{\partial x}dx+\lambda_{2}\frac{\partial F(v)}{\partial y}dy\\ =\int_{\left(0,0\right)}^{\left(x,R-\sqrt{R^{2}-x^{2}}\right)}\lambda_{2}\frac{\partial F(v)}{\partial x}dx+\lambda_{2}\frac{\partial F(v)}{\partial y}dy+\int_{\left(x,R-\sqrt{R^{2}-x^{2}}\right)}^{\left(x,y\right)}\lambda_{2}\frac{\partial F(v)}{\partial x}dx+\lambda_{2}\frac{\partial F(v)}{\partial y}dy\\ =\int_{R-\sqrt{R^{2}-x^{2}}}^{y}\lambda_{2}\frac{\partial F(v)}{\partial x}dx+\lambda_{2}\frac{\partial F(v)}{\partial y}dy=\frac{\lambda_{2}y^{\prime }}{y}F(v) \end{array}$$ (A10)

The right side of Eq A10 can be equated to the right side of Eq A9 to obtain

$$w(x,y)=\frac{y^{\prime }\lambda_{2}}{y}F(v)+{\left[\frac{1}{q}+\frac{q-1}{q}\lambda_{1}\right]}^{\frac{q}{q-1}}$$ (A11)

Substituting Eqs 23 and A3 into Eq A11 gives the expression of velocity distribution

$$v=\frac{1}{\lambda_{2}}\left\{{\left[\lambda_{1}^{q/(q-1)}+\frac{y^{\prime }\lambda_{2}F(v)}{y}{\left(\frac{q}{q-1}\right)}^{q/(q-1)}\right]}^{1-1/q}-\lambda_{1}\right\}$$ (A12)

Eq A12 is the 2D velocity distribution equation in partially-filled circular pipes based on the Tsallis wavelet entropy.

## Supporting Information

S1 Fig Experimental Equipment. The moving accuracy of 3D traversing mechanism is 0.01mm.
(TIF)

S2 Fig Schematic diagram of measurement reference point. Taking the intersection point of fluid section and axial center line as the reference point. The determining of reference point must be carried out in waterless condition.
(TIF)

S3 Fig Schematic diagram of the measuring path. Take 50% depth ratio for example.
(TIF)

S4 Fig Schematic diagram of the initial measurement point. Take 50% depth ratio for example. (a) The position of initial measurement point. In actual fluid flow, the initial measurement point is not on the water surface. (b) The data distribution of the initial measurement point.
(TIF)

S5 Fig The distribution of experimental data of single point. The velocity of each point was measured ten thousands times, and some unreasonable points were deleted according to the hydraulics knowledge, and then the average value is taken as the final result. In the figure, the symbols “-” only represent the direction.
(TIF)

S1 File The experimental data of first measurement point at the condition of 70% depth ratio. (XLS)

S2 File The experimental data of two-dimensional velocity distribution at the condition of 70% depth ratio. (XLS)
