# Supplementary figures and images for: Analysis of the Velocity Distribution in Partially-Filled Circular Pipe Employing the Principle of Maximum Entropy
Source: PLoS One. 2016 Mar 17;11(3):e0151578. doi: 10.1371/journal.pone.0151578 (PMC4795656; doi:10.1371/journal.pone.0151578)

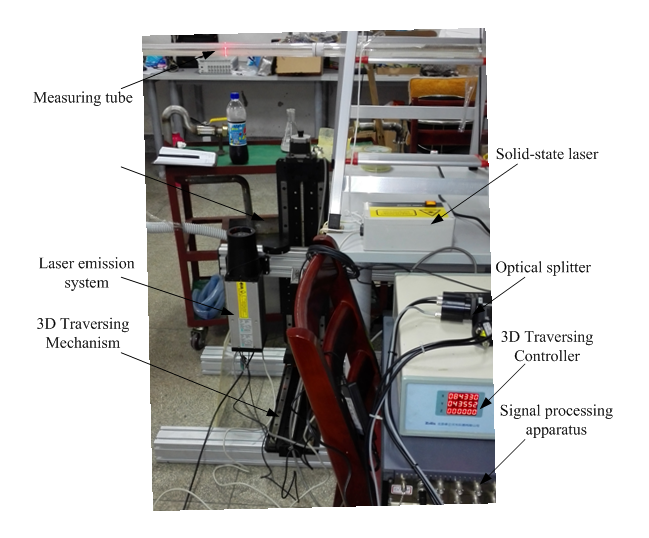

Supplement: S1 Fig — The moving accuracy of 3D traversing mechanism is 0.01mm. (TIF) [file pone.0151578.s001.tif]

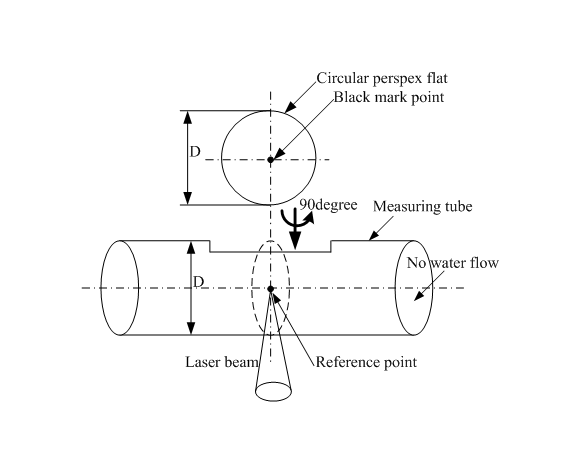

Supplement: S2 Fig — Taking the intersection point of fluid section and axial center line as the reference point. The determining of reference point must be carried out in waterless condition. (TIF) [file pone.0151578.s002.tif]

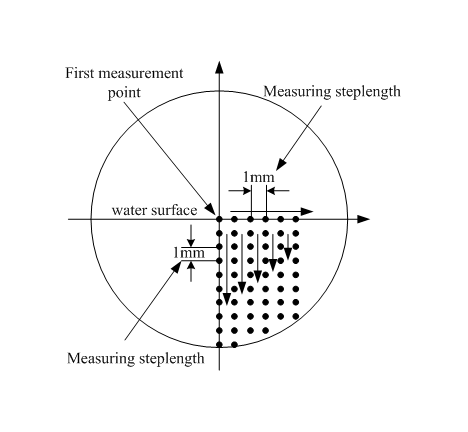

Supplement: S3 Fig — Take 50% depth ratio for example. (TIF) [file pone.0151578.s003.tif]

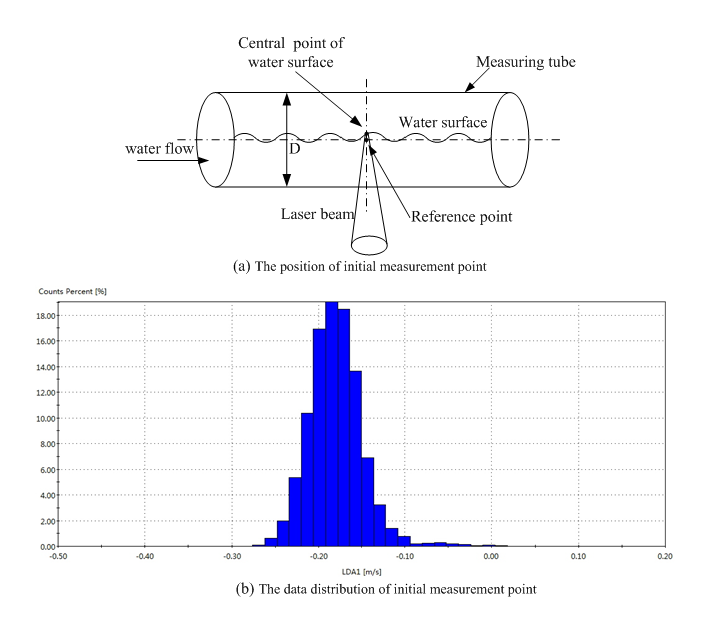

Supplement: S4 Fig — Take 50% depth ratio for example. (a) The position of initial measurement point. In actual fluid flow, the initial measurement point is not on the water surface. (b) The data distribution of the initial measurement point. (TIF) [file pone.0151578.s004.tif]

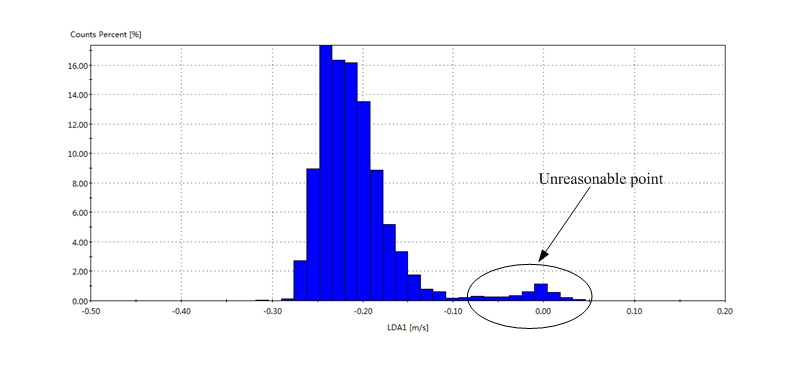

Supplement: S5 Fig — The velocity of each point was measured ten thousands times, and some unreasonable points were deleted according to the hydraulics knowledge, and then the average value is taken as the final result. In the figure, the symbols “-” only represent the direction. (TIF) [file pone.0151578.s005.tif]
